# Post-drought Resilience After Forest Die-Off: Shifts in Regeneration, Composition, Growth and Productivity

**DOI:** 10.3389/fpls.2018.01546

**Published:** 2018-10-25

**Authors:** Antonio Gazol, J. Julio Camarero, Gabriel Sangüesa-Barreda, Sergio M. Vicente-Serrano

**Affiliations:** ^1^Instituto Pirenaico de Ecología (IPE-CSIC), Zaragoza, Spain; ^2^Departamento de Ciencias Agroforestales, EU de Ingenierías Agrarias, Universidad de Valladolid, Soria, Spain

**Keywords:** dendroecology, drought stress, Normalized Difference Vegetation Index, resilience, tree recruitment

## Abstract

A better understanding on the consequences of drought on forests can be reached by paying special attention to their resilience capacity, i.e., the ability to return to a state similar to pre-drought conditions. Nevertheless, extreme droughts may surpass the threshold for the resilience capacity triggering die-off causing multiple changes at varying spatial and temporal scales and affecting diverse processes (tree growth and regeneration, ecosystem productivity). Combining several methodological tools allows reaching a comprehensive characterization of post-drought forest resilience. We evaluated the changes in the abundance, regeneration capacity (seedling abundance), and radial growth (annual tree rings) of the main tree species. We also assessed if drought-induced reductions in growth and regeneration of the dominant tree species scale-up to drops in vegetation productivity by using the Normalized Difference Vegetation Index (NDVI). We studied two conifer forests located in north-eastern Spain which displayed drought-induced die-off during the last decades: a Scots pine (*Pinus sylvestris*) forest under continental Mediterranean conditions and a Silver fir (*Abies alba*) forest under more temperate conditions. We found a strong negative impact of a recent severe drought (2012) on Scots pine growth, whereas the coexisting *Juniperus thurifera* showed positive trends in basal area increment (0.02 ± 0.003 cm^2^ yr^-1^). No Scots pine recruitment was observed in sites with intense die-off, but *J. thurifera* and *Quercus ilex* recruited. The 2012 drought event translated into a strong NDVI reduction (32% lower than the 1982–2014 average). In Silver fir we found a negative impact of the 2012 drought on short-term radial growth, whilst long-term growth of Silver fir and the coexisting *Fagus sylvatica* showed positive trends. Growth rates were higher in *F. sylvatica* (0.04 ± 0.003 cm^2^ yr^-1^) than in *A. alba* (0.02 ± 0.004 cm^2^ yr^-1^). These two species recruited beneath declining and non-declining Silver fir trees. The 2012 drought translated into a strong NDVI reduction which lasted until 2013. The results presented here suggest two different post-drought vegetation pathways. In the Scots pine forest, the higher growth and recruitment rates of *J. thurifera* correspond to a vegetation shift where Scots pine is being replaced by the drought-tolerant juniper. Conversely, in the Silver fir forest there is an increase of *F. sylvatica* growth and abundance but no local extinction of the Silver fir. Further research is required to monitor the evolution of these forests in the forthcoming years to illustrate the cumulative impacts of drought on successional dynamics.

## Introduction

A significant increase in air temperature has been recorder from the early 1980s to the 2010s over Southern Europe, particularly affecting the Mediterranean area (IPCC, 2014; Spinoni et al., 2017). These drying trends have been more marked from winter to summer, i.e., encompassing most of the growing season of the affected species in spring and thus reducing forest productivity and tree vitality (Ciais et al., 2005). In drought-prone areas from the Mediterranean region, some forests are showing die-off events and increased mortality rates as a result of warmer conditions, increased vapor pressure deficit, and more frequent or longer dry spells (Sarris et al., 2007; Linares and Camarero, 2012; Sánchez-Salguero et al., 2012; Camarero et al., 2015). Such chronic and rapid climate-driven disturbances may lead to no-analog situations causing forest disequilibrium (Anderegg et al., 2012), which requires efforts to assess post-drought resilience capacity (Gazol et al., 2018b), and also to better predict die-off impacts on forest dynamics and associated ecosystem services (Anderegg et al., 2016; McDowell et al., 2018). Thus, it is necessary to identify forest states preceding and following drought-driven die-off and to characterize the potentially non-linear transitions separating those states (Cobb et al., 2017).

Drought-triggered die-off usually induces higher tree mortality rates which can trigger vegetation shifts and accelerate successional dynamics (Breshears et al., 2005; Suarez and Kitzberger, 2008; Suarez and Sasal, 2012). Two extreme scenarios after drought-induced mortality can be expected: (1) the persistence of the previously dominant tree species through the survival of most of its adults or their regeneration from the seed and/or seedling bank (Suarez and Lloret, 2018); or (2) the replacement of less drought-resistant species by more resistant species including changes in the functional types prevailing at the community (e.g., shifts from a forest to a scrubland or from gymnosperms to angiosperms; see Gazol et al., 2017c). In a recent review, almost half of the evaluated case studies showed no change in the dominant vegetation type indicating a high post-drought resilience of forests affected by die-off (Martínez-Vilalta and Lloret, 2016). Therefore, this lack of widespread drought-induced forests shifts can be explained by demographic compensation which improves resilience and allows forest structure and composition to be maintained (Lloret et al., 2012). For example, growth decline and increased tree mortality after a drought may be compensated by enhanced recruitment and growth improvement of survivors due to the release of competition for limiting resources such as light and water (Madrigal-González et al., 2017). Alternatively, drought-related die-off may be a too patchy or diffuse disturbance as compared with other more severe or widespread natural (e.g., fire, landslides) and human disturbances so as to trigger vegetation shifts (Anderegg et al., 2016).

Past legacies can also influence how die-off in drought-induced forests is displayed. First, recurrent or more severe droughts could produce a cumulative effect by weakening trees, reducing their growth rates, rising mortality rates and diminishing forest post-drought resilience capacity (Bigler et al., 2006; Peltier et al., 2016; Gazol et al., 2018a). Second, in the case of historically managed ecosystems as Mediterranean forests, die-off could be predisposed by past use leading to a negative selection of slow-growing trees more vulnerable to recent droughts (Camarero et al., 2011; Sangüesa-Barreda et al., 2015). These historical influences require retrospective approaches and prospective monitoring of changes in composition, growth and mortality through time before and after forest die-off (e.g., Suarez and Lloret, 2018). In this sense, annual-growth rings provide a mid-term time perspective on how trees grow and respond to drought and satellite images could provide a picture of how a proxy of productivity and vegetation activity such as the Normalized Difference Vegetation Index (NDVI) responds to drought and potential changes in forest structure through time (Gazol et al., 2018b). Lastly, the combination of several methodological approaches to evaluate shifts in forest composition after drought-induced dieback further supports the novelty and highlights the importance of this study with respect to previous papers (e.g., Camarero et al., 2015). In this sense, multiple methodological tools may provide comprehensive characterizations of post-drought forest resilience. These complementary measures can help answering if drought-induced reductions in growth and regeneration of the dominant tree species scale-up to drops in vegetation productivity.

Here we evaluate the responses of different forest components to an extreme drought in two conifer forests subjected to contrasting climate conditions and displaying declining growth trends during the last decades. In particular, we study how forest productivity, tree growth and regeneration changed in the short (3 years) and long terms (30 years). The short-term response was evaluated after an extreme drought that affected most of the Iberian Peninsula in 2012 and triggered forest die-off. The long-term trend was evaluated by considering tree growth (annual-growth rings) and ecosystem productivity (NDVI) over the last three decades (1980-present). We compared these responses in two forests located in north-eastern Spain, a Scots pine (*Pinus sylvestris*) forest subjected to continental and dry conditions and a Silver fir (*Abies alba*) forest subjected to temperate and wetter conditions. These two species displayed marked growth declines as a consequence of the occurrence of warming trends and related droughts during the last decades (Camarero et al., 2015). We aim to disentangle whether warming trends together with severe drought induced a change in forest composition, growth and productivity by accelerating a replacement of drought-vulnerable with drought-tolerant species.

## Materials and Methods

### Study Sites and Tree Species

We studied a Scots pine (*P. sylvestris* L.) and a Silver fir (*A. alba* Mill.) forest, both located in Aragón, north-eastern Spain (Table 1). These two forests are situated near the southernmost distribution limit of both species in Europe. The Silver fir forest is located in the Spanish central Pyrenees (Paco Ezpela, Ansó) where temperate and wet conditions prevail (see Table 1). Here soils are basic (pH = 6.8), loamy Cambisols with a high proportion of lime (41%) and clay (13%; Gazol et al., 2018a). Despite this forest present signs that intense logging activity was undertaken in the past such as the presence of stumps and wood trails (Sangüesa-Barreda et al., 2015), most of these management activities ceased in the 1970s (Camarero et al., 2015). The Scots pine forest is located in the Iberian System (El Carrascal, Corbalán) and it is subjected to a continental Mediterranean climate. It presents acid (pH = 7.3) Cambisol soils with a relatively high proportion (76%) of sand (Gazol et al., 2018a). This forest presents no evident signs of human management during the last 50 years (Camarero et al., 2015).

**Table 1:** Characteristics of the studied Silver fir and Scots pine forests showing die-off and mortality after the 2012 drought.

Feature or variable	Silver fir	Scots pine
Site name	Paco Ezpela	El Carrascal
Latitude (N)	42° 44′ 28″	40° 26′ 32″’
Longitude (W)	0° 49′ 38″	0° 59′ 16″
Elevation (m a.s.l.)	1120	1285
Aspect	NE	NW
Slope (°)	35	25
Mean annual temperature^§^ (°C)	9.5	12.0
Mean annual precipitation^§^ (mm)	1153	371
Difference between annual precipitation and reference evapotranspiration from previous October up to September (mm)^§^	611 ± 23	-310 ± 38
Difference between annual precipitation and reference evapotranspiration in the 2012 hydrological year, from October 2011 to September 2012 (mm)^§^	-536	-1043
Diameter at 1.3 m (cm)^#^	37.3 ± 1.2	27.3 ± 1.0
Height (m)	24.0 ± 0.5	8.5 ± 0.3
Mortality in 2012 (%)^#^	46	28
Mortality in 2015 (%)^#^	50	71
Mortality in 2017 (%)^#^	60	94
Basal area (m^2^ ha^-1^)	15.0	8.0
Main woody plant species	*Abies alba, Fagus sylvatica, Crataegus monogyna, Corylus avellana, Ilex aquifolium, Acer opalus, Sorbus aucuparia, Buxus sempervirens, Hedera helix*	*Pinus sylvestris, Juniperus thurifera, Quercus ilex, Quercus faginea, Pinus nigra* subsp. *salzmannii*, *Juniperus phoenicea, Juniperus oxycedrus*


**Values are means ± SE. ^§^ Climate data for the Silver fir and Scots pine forests were taken from the Jaca (42° 34′ N, 0° 33′ W, 818 m, located at 30 km from the study forest) and Teruel (40° 21′ N, 1° 06′ W, 915 m, located at 15 km from the study forest) meteorological stations, respectively. ^#^Based on the annual monitoring of vigor proxies (e.g., crown transparency, bud and needle production) and tree death in 35 adult trees of the main species at each site.**

Several investigations performed in these forests suggest that growth decline in the Silver fir forest started after the 1986 drought that affected the Pyrenees, whereas growth decline in the Scots pine forest started in the early 1980s as a consequence of a climate shift toward warmer and dryer conditions (Camarero et al., 2018). Moreover, the two study forests showed a marked growth decline and increased mortality as a consequence of the severe winter-spring drought that occurred in 2011–2012 (Trigo et al., 2013) and affected most Spain (Camarero et al., 2015). For many trees, tree growth decline resulted in rapid death (see more details in Table 1).

### Evaluation of Post-drought Tree Regeneration and Recruitment

In 2012, a total of 38 dominant trees were randomly selected and permanently marked with a balanced number of declining and non-declining individuals in each forest (Camarero et al., 2015). We measured the size (Dbh, diameter at breast height measured at 1.3 m; height) and estimated the crown transparency (defoliation degree) using binoculars of each tree (Dobbertin, 2005). Tree crown transparency, tree vigor and mortality were monitored in 2012 and annually from 2015 to 2017. Declining and non-declining individuals were defined as those showing crown transparency above or below the 50% threshold, respectively.

To account for the effects of local neighborhood on tree performance (Zambrano et al., 2017) the neighborhood (basal area) of each of the sampled trees was characterized in 2015. We measured the Dbh and identified the species of all stems higher than 1.3 m and located within a circular plot of radius 7.6 m centered on the sampled focal tree. A similar radius was used in the two forests to avoid changes due to vegetation structure and composition. These values were used to calculate the basal area and the density of the main species of woody plants in the community below declining or recently dead and non-declining trees. We also quantified the regeneration capacity of the different woody species by counting the number of recruits (seedlings and saplings with height < 0.25 m) located in the forest understory. Particularly, we quantified the presence of recruits from different species in 100 squares (size 25 cm × 25 cm) randomly placed in a representative area of the forest floor. These representative areas were selected to reflect the general conditions of the forest across an area of 0.1 ha, i.e., the number of squares was proportionally located according to the area with or without die-off signs (dead or declining trees showing high needle shedding). The evaluation of tree regeneration was repeated yearly from 2015 to 2017 in the same area of the two forests.

### Growth Data: Dendrochronological Methods

In order to quantify the radial-growth patterns and trends of the two dominant conifers and the most important co-dominant species, we sampled several trees using dendrochronological methods. All the sampled individuals were apparently healthy trees, with low crown defoliation, to avoid potential bias in growth as a consequence of the decline of the dominant tree species. Specifically, we sampled *P. sylvestris* (*n* = 35 trees) and *Juniperus thurifera* (*n* = 15 trees) in the Scots pine forest and *A. alba* (*n* = 36 trees) and *Fagus sylvatica* (*n* = 49 trees) in the Silver fir forest. The low number of sampled *J. thurifera* individuals was due to the low abundance of mature individuals of this species in the Scots pine forest (Table 2). Two increment cores were taken from each tree at 1.3 m and perpendicularly to the maximum slope using Pressler increment borers in late 2015.

**Table 2:** Values of basal area (in m^2^ ha^-1^; means ± SE) of the most abundant tree species for neighborhoods in declining or recently dead trees (die-off) or non- declining trees (no die-off).

Forest type	Tree species	Neighborhood type		*t* (*P*)
			
		Die-off	No die-off	
Silver fir	*Abies alba*	16.1 ± 3.7	13.9 ± 2.6	0.47 (0.66)
	*Fagus sylvatica*	5.1 ± 0.5	3.4 ± 0.4	**2.44** (0.03)
	*Crataegus monogyna*	0.4 ± 0.1	0.3 ± 0.1	1.06 (0.23)
Scots pine	*Pinus sylvestris*	7.8 ± 1.4	7.7 ± 1.6	0.03 (0.98)
	*Juniperus thurifera*	2.5 ± 0.3	2.2 ± 0.4	0.74 (0.48)
	*Quercus ilex*	2.0 ± 0.4	0.2 ± 0.1	**4.71** (0.04)


*The measurements were taken in 2015 (3 years after the 2012 drought). The last column shows the comparison between variables based on *t*-tests with their corresponding *P*-values. Significant *t*-values (*P* < 0.05) are indicated with bold characters.*

These cores were mounted and air dried in the laboratory. After that, the cores were sanded with progressively finer sandpaper until tree rings were visually recognizable. Ring widths were measured at 0.01 mm resolution using the LINTAB measuring device (Rinntech, Heidelberg, Germany). Visual cross-dating was performed and checked using the program COFECHA (Holmes, 1983). Tree ring widths were transformed to basal area increment (BAI*t*) using the following formula:

$$BAIt=\pi \left(R_{t}^{2}-{R_{t-1}}^{2}\right)$$

where *R_t_* is the radius of the ring formation year and *R*_*t*-1_ is the radius of the year preceding the ring formation. We also calculated the relative basal area increment as the ratio between the observed BAI divided by maximum BAI of each species to compare post-drought growth responses between species.

### Remote-Sensing Data: NDVI

To quantify the temporal variability in aboveground forest gross-primary productivity in each forest we used 1.1 km^2^ spatial resolution bi-weekly series of the Normalized Difference Vegetation Index (NDVI) over the period 1982–2014. NDVI was obtained from daily NOAA-AVHRR images from 1981 to 2015. These images were subjected to a complete calibration, quality control, geometric matching, cloud removal and topographic correction (Hantson and Chuvieco, 2011). After the calculation of the NDVI, the daily images were aggregated into semi-monthly composites by means of the Maximum Value Composite (MVC) technique. The quality and accuracy of this dataset was verified, given its strong temporal coherence in comparison to other available datasets at lower spatial resolution (Martín-Hernández et al., 2017). In this study we selected the two 1.1 km^2^ pixels that corresponded to the two study forests.

### Statistical Analyses

We compared the neighborhood below declining and non-declining trees in each forest. Particularly, we tested whether some of the most abundant woody plant species were more or less abundant below declining and non-declining trees. A *t*-test was applied to study whether there were significant differences in the abundance of woody plants below declining and non-declining trees.

To compare tree regeneration below declining and non-declining trees, we used the Cramér’s *V* statistic (Agresti, 2013). This statistic is the square root of χ^2^ divided by sample size (*n*) times *m*, which is the smaller of *r* (number of rows - 1) or *k* (number of columns - 1):

$$V=\sqrt{\frac{\chi^{2}}{n\times m}}$$

Cramér’s *V* ranges from 0 to 1 corresponding to dissociation (no association) or complete association between the compared variables, respectively (Agresti, 2013).

We assessed the short- and long-term growth trends of the dominant tree species in each forest (*P. sylvestris* and *A. alba*) and the two main co-dominant tree species (*J. thurifera* and *F. sylvatica*, respectively). In the short-term, we studied whether the radial growth (BAI) 3 years after the 2012 drought (period 2013–2015) differed from that observed during the drought and 3 years before (period 2009–2011). We selected the 3-year period based on previous studies showing that it adequately captured the post-drought response (Gazol et al., 2017a,b, 2018b). Comparisons of growth between these periods were performed using ANOVA followed by Tukey-HSD tests when significant differences in growth between the pre-drought and post-drought periods was observed.

In the long-term, we studied the BAI trends of the main tree species (*P. sylvestris*, *J. thurifera*, *A. alba*, and *F. sylvatica*) for the common period 1982–2015. We fitted linear mixed-effect models using BAI as response variable (Zuur et al., 2010). We run a separate model for each tree species (*P. sylvestris, J. thurifera*, *F. sylvatica*, and *A. alba*). To represent the linear growth trend, we included calendar year as a predictor variable; a positive effect of year indicates growth enhancement whereas a negative effect indicates growth decrease. Tree radial growth depends on tree ontogeny and Dbh (Gazol et al., 2017b). Therefore, we included the estimated tree age and the Dbh reconstructed back in time as covariates in the model. Tree age at 1.3 m was estimated by counting the number of rings in the oldest core. In cores without pith, pith-offset estimations were used to calculate the number of missing rings by fitting a geometric pith locator. Since the BAI of each tree represents repeated measures across an individual, we include tree identity as random factor. To control for the potential temporal autocorrelation, we also included a first-order autocorrelation structure (AR1). To linearize growth measures and achieve normality assumptions, we log transformed [*log(x + 1)*] BAI prior to the analyses (Zar, 2010).

To identify the set of covariates that better explained the observed BAI trends, we applied a multi-model inference approach based on information theory (Burnham and Anderson, 2002). We ranked all potential models according to the second-order Akaike information criterion (AICc) and selected as the best model that showing the lowest AICc value and largest Akaike weight (which represents the relative probability that the selected model is the best one). The different competing models were ranked according to the ΔAICc (AICc differences between the model selected and the rest of remaining models).

To compare the growth trends between species, we fitted linear mixed-effect models using BAI as response variable (Zuur et al., 2010) and including calendar year, tree age and dbh and species identity as explanatory variables. To account for different growth trajectories between species, we included an interaction between calendar year and species identity. We also considered potential interactions between tree age and species identity and Dbh and tree species. A multi-model inference approach based on information theory (Burnham and Anderson, 2002), was used to select the most parsimonious model (lowest AICc value and largest Akaike weight). When significant interactions between calendar year and species identity were found, we performed least-squares means based on and Tukey HSD tests to assess the differences between tree species (Bretz et al., 2010).

In order to detect changes in aboveground productivity along time in each forest, we modeled NDVI for the period 1982–2014 using Generalized Additive Models (Wood, 2006). These are flexible semiparametric models that allow modeling the response variable as different smooth functions of a set of explanatory covariates. Clearly, the NDVI bi-weekly data contain a strong year cycle, but it is less clear whether it presents long term trends. Thus, we modeled how NDVI varies within and between years along the entire study period. To represent variations in NDVI bi-weekly data within a year, we used a cyclic cubic regression spline since a cyclic smooth is recommended for repeated patterns (Wood, 2006). To model the long-term variation in NDVI along the entire study period we used thin plate splines. A first-order correlation structure (AR1) was included to account for the temporal autocorrelation in NDVI.

All statistical analyses were performed in the R environment (R Core Team, 2017). The ‘*dplR*’ package was used to convert tree-ring width series into BAI (Bunn et al., 2016). The *lme* function of the ‘*nlme*’ package was used to fit the linear mixed-effects models (Pinheiro et al., 2014) and the ‘*MuMIn*’ package was used to perform the multi-model selection (Barton, 2012). The *gam* function of the ‘*mgcv*’ package was used to fit the generalized additive models (Wood, 2006).

## Results

### Mortality, Forest Structure and Post-drought Tree Recruitment

**FIGURE 1:**
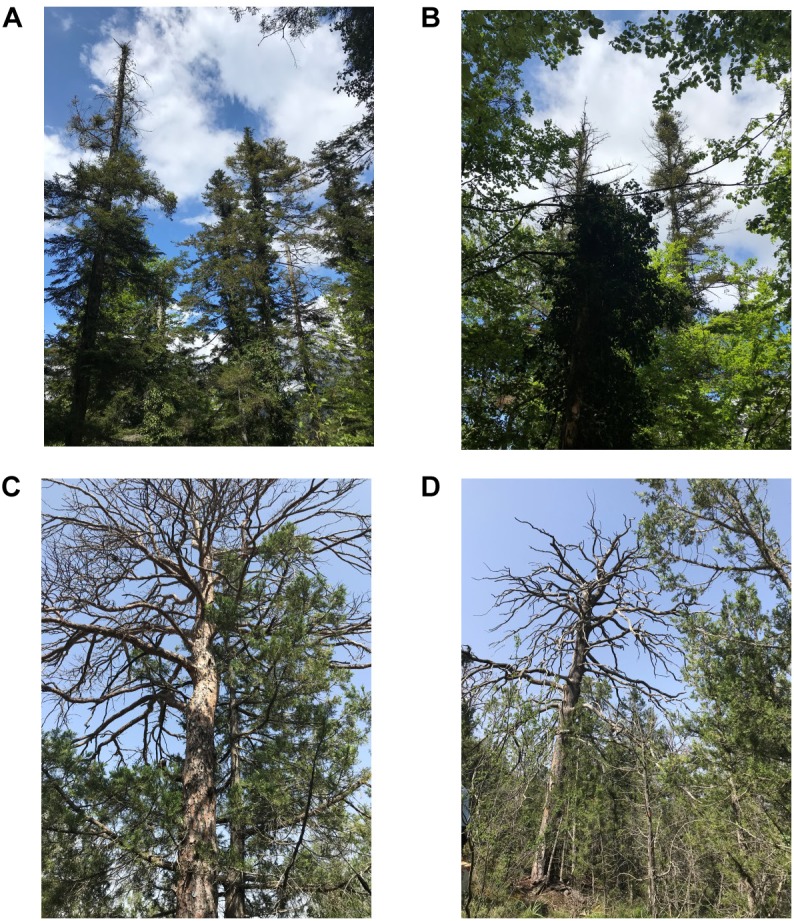
Views of declining and dead Silver fir **(A,B)** and Scots pine **(C,D)** trees.

Mortality from 2012 to 2017 was higher and increased more in the Scots pine forest (mean ± *SE* = 52.0 ± 4.2%; mean rate, 22.0% yr^-1^) than in the Silver fir forest (64.3 ± 19.3%; mean rate, 4.7% yr^-1^; Table 1). We found a high abundance of *P. sylvestris* and *A. alba* individuals in the neighborhood of the focal *P. sylvestris* and *A. alba* trees sampled in each forest, respectively (Table 2). This means that the neighborhood of either declining or non-declining trees is mainly composed by conspecifics in both forests. Nevertheless, we found differences in the abundance of other species in the neighborhood of declining and non-declining individuals. In the *P. sylvestris* forest, *Quercus ilex* was significantly more abundant below declining than non-declining trees. Similarly, *F. sylvatica* was more abundant below declining than below non-declining *A. alba* trees (Figures 1A,B and Table 2). In the *P. sylvestris* forest, *J. thurifera* was abundant (Figures 1C,D), but its basal area did not differ between declining and non-declining trees.

**FIGURE 2:**
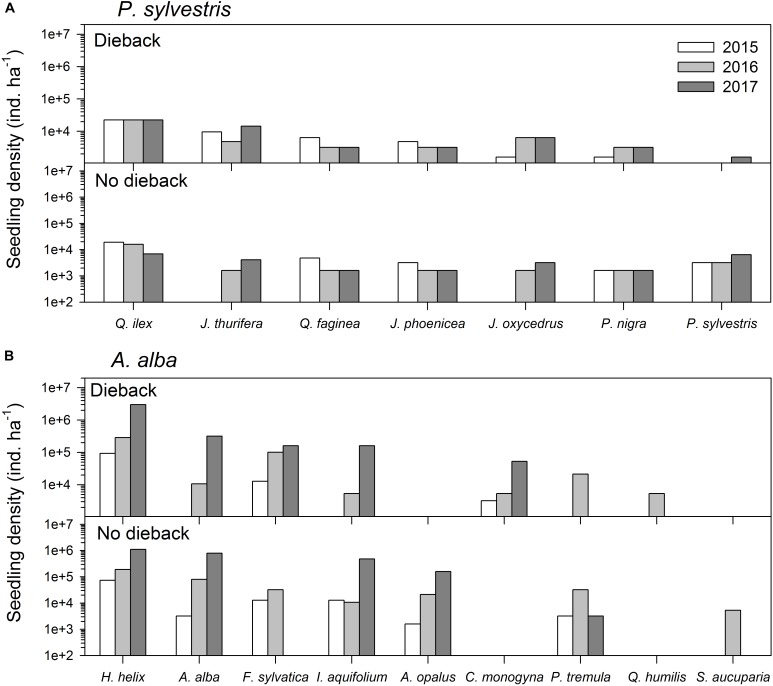
Comparison of recruitment of the main woody plant species three (2015), four (2016), and five (2017) years after the drought-induced die-off episode started affecting the study **(A)** Scots pine (*Pinus sylvestris*) and **(B)** Silver fir (*Abies alba*) forests. See a list of the main woody plant species found at each site in Table 1.

Regarding tree recruitment, Cramér’s *V* values indicated that regeneration below declining and non-declining trees differed in 2015 but tended to be more similar in 2017, particularly in the *A. alba* forest (Figure 2 and Table 3). Overall, species recruited below declining and non-declining trees differed more in the *P. sylvestris* (mainly in 2016) than in the *A. alba* forest. The species most abundantly recruiting in the *P. sylvestris* forest were *Q. ilex*, *J. thurifera*, and *Q. faginea*, whilst *Hedera helix*, *A. alba*, and *F. sylvatica* were the main recruiting species in the *A. alba* forest (Figure 2). In 2015, no *A. alba* recruit was found below declining conspecific trees, whereas we did not observe any *P. sylvestris* recruit below conspecific declining trees neither in 2015 nor in 2016 (Supplementary Figure S1).

**Table 3:** Cramér’s *V* tests comparing the seedlings and saplings density of the main woody species recruited in sites with or without declining or recently dead trees due to drought-induced die-off.

Year	Forest type	
	
	Silver fir	Scots pine
2015	0.34	0.32
2016	0.41	0.31
2017	0.52	0.40


**Comparisons were made three (2015) to five (2017) years after die-off and tree mortality of the two dominant tree species in each forest (Silver fir, Scots pine) started in 2012. Cramér’s V ranges from 0 to 1 corresponding to lack of or complete association between variables, respectively. In all cases P-values are lower than 0.0001 for the corresponding χ^*2*^ statistics*.*

### Radial Growth Patterns at Long- to Short-Term Scales

**FIGURE 3:**
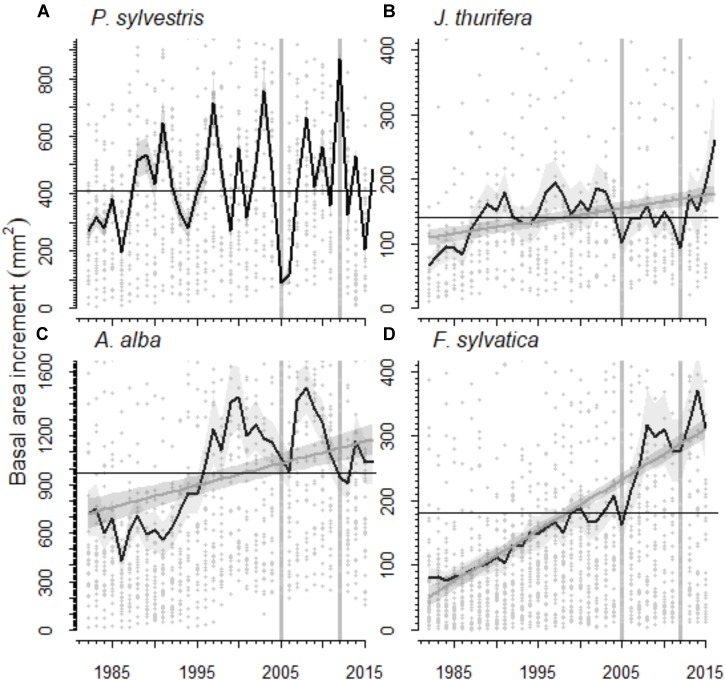
Basal area increment (BAI) of the species studied in the **(A,B)** Scots pine (**A**, Scots pine, *P. sylvestris*; **B**, Spanish juniper, *Juniperus thurifera*) and **(C,D)** Silver fir (**C**, Silver fir, *A. alba*; **D**, *Fagus sylvatica*, beech) forests for the period 1982–2015. The black line represents the average BAI for each species and the shaded gray area around it the standard error of the mean. The gray line represents the trend in BAI according to the linear mixed-effect models and the shaded gray area around it the standard error of the prediction (in Scots pine no significant BAI trend was observed). Gray dots represent individual BAI measures. Black lines indicate the average BAI for the period 1982–2015. Vertical gray lines highlight the 2005 and 2012 droughts.

**FIGURE 4:**
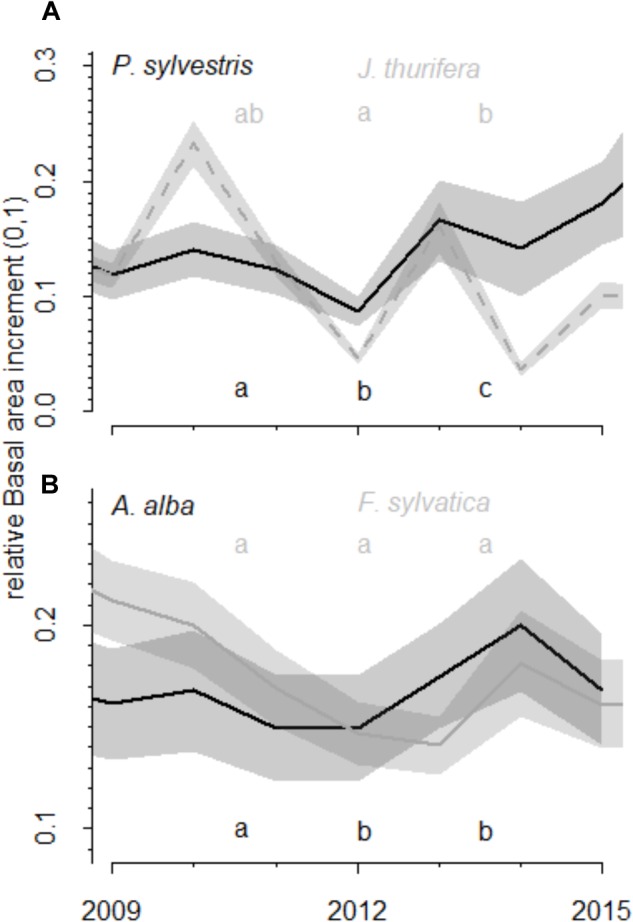
Comparison of growth values (relative basal area increment) before, during and after the drought. The relative basal area increment was calculated as observed BAI divided by maximum BAI of each species. The relative basal area increment observed before (2009–2011), during (2012) and after the 2012 drought (2013–2015) event in the **(A)** Scots pine and **(B)** Silver fir forests is shown. Different colors represent different species (black and gray). Lines represent mean BAI and the shaded areas the standard errors. Letters indicate the existence of significant differences between growing periods according to Tukey-HSD tests.

The radial growth (BAI) of the main tree species presented a marked decline as a consequence of the 2012 drought (Figures 3, 4). All species showed marked growth reductions in response to the severe 2005 and 2012 droughts (Figure 3). A marked growth decline as a consequence of the 2012 drought was significant in all species excepting *F. sylvatica*. Long-term growth trends differed significantly between species (Figure 3 and Table 4). In the *P. sylvestris* forest, the model for *P. sylvestris* BAI explained 56% of the variation in its growth trends and did not include the effect of year, indicating no clear growth trends. Conversely, the model for *J. thurifera* presented a significant positive trend (0.02 ± 0.003 cm^2^ yr^-1^) indicated by the significant effect of year (Table 4). This model also included a significant effect of tree dbh and accounted for 61% of the variation in *J. thurifera* BAI. In the *A. alba* forest, *A. alba* and *F. sylvatica* presented significant positive trends (significant influence of year, Table 4) but with a stepper slope in the case of *F. sylvatica* (0.04 ± 0.003 cm^2^ yr^-1^) than in *A. alba* (0.02 ± 0.004 cm^2^ yr^-1^). The models accounted for 49 and 76% of the variation in *A. alba* and *F. sylvatica* BAI, respectively. In all models excluding the *F. sylvatica* model, tree age exerted a negative influence on BAI trends, whereas tree Dbh was significantly and positively related to BAI trends for all species (Table 4). These results were consistent with those obtained when considering the four species in a single model (Supplementary Figure S2). The model showed the existence of significant effect of tree Dbh, tree age and calendar year and their interactions with tree species on BAI. This model, which accounted for 81% of the variation in BAI, indicated the existence of significantly different growth trajectories between species (Supplementary Table S2). The *post hoc* analyses showed that *P. sylvestris* presented growth rates lower than the rest of species. The growth rates of *A. alba* and *J. thurifera* did not differ between them. *F. sylvatica* presented the greatest growth rates.

**Table 4:** Results of the linear mixed-effect models selected to study radial-growth trends of the four-tree species (*P. sylvestris*, *J. thurifera*, *A. alba*, and *F. sylvatica*).

Tree species	Tree age	Dbh (diameter at breast height)	Year	ΔAICc	Weight	Pseudo *R*^2^	
						
						Fixed effects	Fixed and partial effects
*P. sylvestris*	-2.26^∗∗^	9.41^∗∗^	–	1.09	0.50	0.46	0.56
*J. thurifera*	-1.63	7.94^∗∗^	5.15^∗∗^	0.44	0.56	0.52	0.61
*A. alba*	-3.16^∗∗^	9.13^∗∗^	4.96^∗∗^	7.19	0.97	0.49	0.49
*F. sylvatica*	–	23.19^∗∗^	13.61^∗∗^	1.04	0.63	0.74	0.76


**Radial growth was quantified as log_*10*_ (*BAI+1*) where BAI is the basal area increment. The t-statistic associated to each variable included in the model is shown. Positive signs indicate a positive relationship whereas negative signs indicate a negative relationship. The ΔAICc, Akaike-weight, and pseudo-R^*2*^ (due to fixed and due to fixed and partial effects, respectively) of each selected model is also provided. Significance of the t statistic is indicated by: ^∗∗^p < 0.01.**

The short-term responses of relative BAI to the 2012 drought showed different patterns for the growth of each species (Figure 4). In the Scots pine forest, *P. sylvestris* growth was significantly higher before (mean BAI was 504.8 ± 42.9 mm^2^) than during (146.0 ± 16.4 mm^2^) and after (314.1 ± 40.9 mm^2^) the drought, and the post-drought growth was also higher than the growth during the drought year. The *J. thurifera* growth was also significantly higher after (172.8 ± 38.1 mm^2^) than during (92.6 ± 13.8 mm^2^) the drought and no differences in growth were detected before (135.7 ± 23.3 mm^2^) and after the drought event. In the *A. alba* forest, *A. alba* growth was significantly lower during (947.0 ± 99.7 mm^2^) and after the drought (1037.6 ± 125.6 mm^2^) than before it (1246.2 ± 131.8 mm^2^). No differences between *F. sylvatica* growth before (294.1 ± 50.8 mm^2^), during (276.1 ± 48.5 mm^2^) and after the drought (333.0 ± 50.8 mm^2^) were observed.

### Changes in Gross Primary Production as Reflected by the NDVI

**FIGURE 5:**
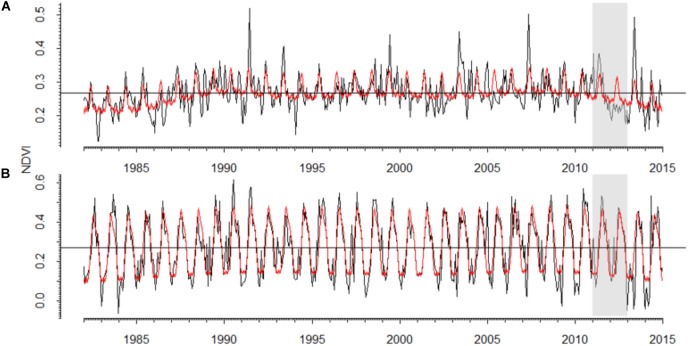
Values of the Normalized Difference Vegetation Index (NDVI) observed for the period 1982–2014 in the studied **(A)** Scots pine and **(B)** Silver fir forests. Black lines represent bi-weekly measures of NDVI and red lines represent the values predicted by generalized additive effect models. The gray boxes highlight the 2012 drought.

In the *P. sylvestris* forest, the NDVI pattern was noisier than in the *A. alba* forest which showed a more marked seasonal pattern (Figure 5 and Supplementary Table S1). The 2012 drought triggered a strong decrease in NDVI in the *P. sylvestris* forest, where predicted NDVI values were 22% higher than observed NDVI values (Figure 5), which were also 32% lower than the observed NDVI values for the entire period. In the *A. alba* forest, no clear differences between predicted and observed NDVI values were observed. Long-term NDVI trends suggest the existence of a lagged decline in NDVI during 2013–2014 in the Silver fir forest (Supplementary Figure S3).

## Discussion

We found evidences showing a potential compositional shift in the two studied forests as a consequence of recent warming trends and the severe droughts, including that in 2012, which caused die-off in both forests. We support this argument on the following facts: (i) recruitment of *P. sylvestris* and *A. alba* was low, null in the case of *P. sylvestris*, below declining trees during two or one of the three monitoring years; (ii) the neighborhood of declining individuals showed an increase in the abundance of other tree species; and (iii) the growth of *P. sylvestris* and *A. alba* decreased as a consequence of drought, at least in the short term, or were less steep than that of coexisting species (*J. thurifera* and *F. sylvatica*, respectively). Based on these findings, we expect that changes in canopy species composition can be observed at local scales in the studied forests during the forthcoming decades. Whether these changes can induce further changes in forest productivity remains an unresolved issue, but we observed a clear reduction in NDVI in the *P. sylvestris* forest which showed the highest mortality rates (Table 1). In this sense, this forest represents an excellent setting to monitor post-drought compositional changes due to high tree diversity, the climatic marginality of this *P. sylvestris* population and a relatively low tree coverage which makes it sensitive to temperature and evapotranspiration increases (Vicente-Serrano et al., 2013).

The resilience of a forest to an extreme drought event mainly depends on the capacity of trees to resist or recover after such severe episode of water shortage (Gazol et al., 2018b). Alternatively, forest resilience is contingent on the capacity of other tree species to become more abundant by recruiting in suitable microsites with favorable soil and microclimatic conditions (Lloret et al., 2012; Hodgson et al., 2015; Redmond et al., 2018). Thus, tree regeneration capacity is the main driver of forest resilience capacity when drought results in the destruction of the vast majority of overstory trees of the dominant tree species in mixed stands (Redmond et al., 2015; Martínez-Vilalta and Lloret, 2016; Redmond et al., 2018; Suarez and Lloret, 2018). In the *P. sylvestris* forest, we found an extremely low presence of *P. sylvestris* seedlings particularly in the neighborhood of declining and dead individuals during three consecutive years after the severe drought (Figure 2). Tree recruitment mainly depends on seed availability, germination capacity and post-drought survival of recruits, and it is well known that drought and climate warming can strongly suppress cone production, reduce seed viability and increase recruit mortality in several conifer species (e.g., Redmond et al., 2012, 2018). In this sense, Galiano et al. (2010) and Vilà-Cabrera et al. (2013) observed a decline of *P. sylvestris* regeneration in some Spanish forests which they attributed to a failure in reproductive success and cone production. The lower seedling density of *P. sylvestris* as compared to the rest of co-occurring species, suggest a greater capacity of the rest of species for seedling establishment at least in declining stands subjected to recent dry spells. In this sense, *P. sylvestris* was among the most drought-vulnerable species of that forest according to its poor regeneration. These results contrast with the *A. alba* forest in which the seedling density of this species did not differ between declining and non-declining sites.

In the *P. sylvestris* forest, we found that in the short-term the radial growth of *P. sylvestris* and *J. thurifera* responded negatively to drought. However, while *J. thurifera* presented a growth enhancement after drought, *P. sylvestris* showed similar growth rates than during drought (Figure 3). Consistently, *J. thurifera* displayed strongly positive growth trends whereas *P. sylvestris* showed no clear trends. Together with the strong *P. sylvestris* mortality observed in this forest (Camarero et al., 2015; Gazol et al., 2018a), these results point to the local extinction of *P. sylvestris* in this site as a consequence of warming trends and recurrent drought during decades. *J. thurifera* is a species perfectly adapted to the Mediterranean continental conditions that prevail in that area which allows this species displaying a great ability to couple either with warm and dry stressing conditions in summer or cold stress in winter (Gimeno et al., 2012; Camarero et al., 2014). Several studies suggest that Fagaceae broadleaf species may replace pine species after disturbances (e.g., Alfaro-Reyna et al., 2018). In this sense, we found a greater abundance of *Q. ilex* below declining *P. sylvestris* individuals than below non-declining individuals, and *Q. ilex* seedlings were fairly abundant below declining and non-declining *P. sylvestris* individuals (Figure 2). Thus, both species, *J. thurifera* and *Q. ilex*, might become dominant if further die-off and mortality affect nearby *P. sylvestris* populations due to warmer and drier conditions.

In the *A. alba* forest, long-term positive growth-trends were found in both, the dominant species and the co-dominant *F. sylvatica*, despite with a stepper slope in the case of beech (Figures 3 and Supplementary Figure S2). In the short term, *A. alba* had a significantly lower growth during and after the drought than before the drought, whereas no changes in growth in response to drought were found in *F. sylvatica* (Figure 4). Many *A. alba* forests in the Pyrenees displayed declining growth trends and die-off after the 1986 severe drought (Camarero et al., 2011; Gazol et al., 2015, 2018a; Sangüesa-Barreda et al., 2015), and in many of these forests declining growth trends have been exacerbated by successive droughts in 2005 and 2012 (Camarero et al., 2018). This is the case of the studied forest, which presents negative growth trends in several *A. alba* individuals and enhanced mortality during the last decades (Camarero et al., 2015, 2018). Conversely, no signs of decline and high mortality have been observed in *F. sylvatica* trees at the same forest (personal observation). In this forest we found a greater growth resilience capacity of *F. sylvatica* and higher long-term growth trends. Moreover, we also observed that *F. sylvatica* trees were more common below declining than non-declining *A. alba* trees, despite no clear patterns in beech regeneration were found. Thus, these results suggest that *F. sylvatica* is favored over Silver fir in the studied forest after the 2012 drought and the subsequent *A. alba* die-off. Such forecasted replacement agrees with the expected successional dynamics (replacement of the conifer by the angiosperm species; cf. Bond, 1989) which have been probably accelerated by drought.

It must be also commented that the studied forests did not include the whole forested area dominated by *P. sylvestris* and *A. alba* in each region. Therefore, we are dealing with local die-off events causing patchy mortality patterns and leading to vegetation shifts in particularly unfavorable sites (Anderegg et al., 2016). Those sites often present the worst conditions for the performance of the dominant tree species such as high aridity, negative selection of slow-growing trees due to past logging and soils with a low ability to infiltrate and hold water (Camarero et al., 2011, 2015; Gazol et al., 2018a). Consequently, the described effects of drought-triggered die-off on tree composition, growth and productivity mainly occur at small to mid-spatial scales (1–100 ha), which allow the affected species growing and surviving in nearby sites with more favorable conditions for their performance thus assuring their regional persistence.

There is a general congruence between tree growth response to drought and primary productivity variation at larger scales (Gazol et al., 2018b). However, whether the observed drying trends in the dominant tree species in the forest scale-up to vegetation productivity at larger scales remains an unanswered question. Semi-monthly NDVI data showed a more seasonal and predictable temporal pattern in the *A. alba* forest than in the *P. sylvestris* forest, probably because of the prevailing climatic conditions: temperate-wet vs. continental and dry Mediterranean conditions (Vicente-Serrano et al., 2013). The two sites showed a marked NDVI reduction as a consequence of the 2012 drought which in the case of the *A. alba* forest lasted until the following year. This is probably due to the well-known dependency of *A. alba* growth on the climatic conditions of the previous year, particularly on the late-summer water balance (Camarero et al., 2011). Unfortunately, the evaluated NDVI series end in 2015 (Martín-Hernández et al., 2017), making difficult to study the consequences of the 2012 drought due to the short post-drought period. Thus, further analyses on longer NDVI series might be required to understand this issue.

In a recent review of vegetation changes associated to drought-induced forest decline, Martínez-Vilalta and Lloret (2016) found evidence of vegetation shifts in only 8 out of 35 cases. From a demographic point of view, drought-induced vegetation shifts should be characterized by the mortality of at least one dominant species and enhanced growth and recruitment of other potential dominant species in the forest (Lloret et al., 2012; Martínez-Vilalta and Lloret, 2016). The results obtained in the *P. sylvestris* forest support this criterion indicated by: (i) the high mortality rates of the dominant species (*P. sylvestris*); (ii) a growth enhancement of the co-dominant species (*J. thurifera*); and (iii) a much greater regeneration success of the co-dominant as compared to the formerly dominant species. Nevertheless, additional time is required to certainly establish that the observed changes are a truly drought-induced vegetation shift as there is a certain period of time in which the former vegetation still has a chance to return to the previous state which could be regarded as community or population inertia (Hughes et al., 2013). However, the results presented here concur with our previous findings indicating that the 2012 drought induced drastic population and community changes in the *P. sylvestris* forest (Camarero et al., 2015). These changes are related to shifts at the ecosystem level as reflected the NDVI data (Gazol et al., 2018b), and in sight of the results presented here may lead to vegetation changes toward communities or populations dominated by more drought-tolerant species or individuals. Our findings illustrate and warn against the local extinction of some tree populations near their southernmost distribution limits as in the *P. sylvestris* forest studied which may become more vulnerable to forecasted aridification trends (Sánchez-Salguero et al., 2017). This is a relevant and urgent concern for researchers and managers which demands mitigation measures such as thinning or considering replacing drought-vulnerable species or individuals by more drought-tolerant species.

## Author Contributions

AG and JC conceived the study. AG, JC, and GS-B designed the field study and collected the data. SV-S provided the NDVI data. AG and JC conducted the analyses. SV-S was in charge of the NDVI data processing. AG wrote the manuscript. All authors contributed to revising the paper.

## Conflict of Interest Statement

The authors declare that the research was conducted in the absence of any commercial or financial relationships that could be construed as a potential conflict of interest.
