# Supplementary material for: Post-drought Resilience After Forest Die-Off: Shifts in Regeneration, Composition, Growth and Productivity
Source: Front Plant Sci. 2018 Oct 25;9:1546. doi: 10.3389/fpls.2018.01546 (PMC6210004; doi:10.3389/fpls.2018.01546)
Supplement: Supplementary file 1 [file Data_Sheet_1.docx]

**Post-drought resilience after forest die-off: shifts in regeneration, composition, growth and productivity**

Antonio Gazol, J. Julio Camarero, Gabriel Sangüesa-Barreda and Sergio M. Vicente-Serrano

**Supporting Information**

**Table S1.** Summary of the Generalized Additive Mixed Models fitted to explain the temporal trend of Normalized Difference Vegetation Index (NDVI) in the studied Scots pine and silver fir forests. For each model, we provide the estimated degrees of freedom (edf) and the *F* statistics associated to each variable as well as the approximate Generalized Cross Validation (GCV) statistic and the R^2^ of the model. Significance level: ** *p* < 0.01.

| Forest type | Biweekly trend | | Long-term trend | | GCV | R^2^ |
| --- | --- | --- | --- | --- | --- | --- |
|  | edf | *F* | edf | *F* |  |  |
| Scots pine | 7.58 | 32.87** | 8.34 | 16.30** | 0.002 | 0.33 |
| Silver fir | 7.47 | 279.38** | 5.38 | 4.67** | 0.005 | 0.74 |

**Table S2.** Results of the linear mixed-effect model selected to study radial-growth trends of the four-tree species together (*P. sylvestris*, *J. thurifera*, *A. alba*, *F. sylvatica*). Radial growth was quantified as log_10_ (BAI) where BAI is the basal area increment. The *F* statistic associated to each variable and the interactions included in the model are shown. The ΔAICc, Akaike-weight, and pseudo-R^2^ (due to fixed and due to fixed and partial effects, respectively) of each selected model is also provided. Significance of the *F* statistic is indicated by: ** *p* < 0.01.

| Tree age | Tree age* species | Dbh (diameter at breast height) | Dbh* species | Year | Year * species | ΔAICc | Weight | pseudo R^2^ |
| --- | --- | --- | --- | --- | --- | --- | --- | --- |
| 26.33** | 2.30 | 1856.90** | 84.00** | 285.38** | 39.88** | 1.37 | 0.67 | 0.81 (0.79) |

**Figure S1.** Recruitment of woody plant species in areas with or without die-off three (2015), four (2016) and five (2017) years after the drought-induced die-off episode (2012) in the studied silver fir and Scots pine forests.


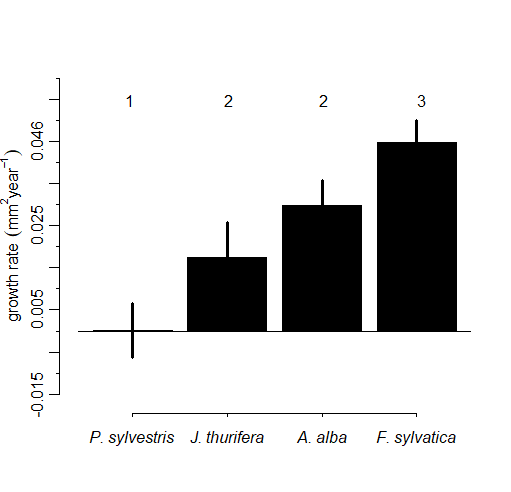


**Figure S2.** Partial effects of year on the growth (BAI) of each tree species according to the post-hoc analyses performed after the linear mixed-effect model including all species together (see Table S2). For each species, the bars represent the trend in growth per year and the segments the standard error for the effect. Different numbers indicate the existence of significant (*p* < 0.05) differences in growth trend between species.


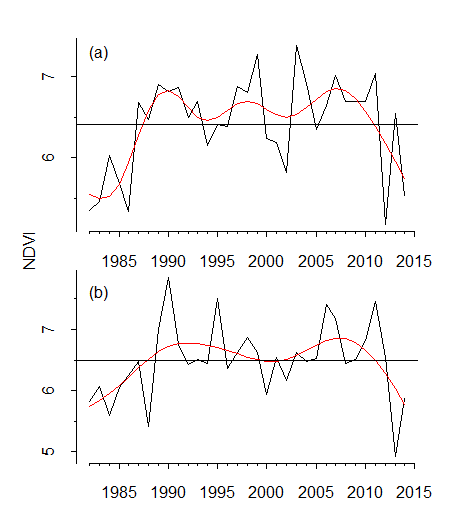


(a)

(b)

**Figure S3.** Annual values of Normalized Difference Vegetation Index (NDVI) calculated for the period 1982-2014 in the studied (a) Scots pine and (b) silver fir forests. The black lines represent the observed NDVI values and the red lines show the predicted trend according to the fitted Generalized Additive Mixed Models.
